# Iodine based radiopacity of experimental blood clots for testing of mechanical thrombectomy devices

**DOI:** 10.2478/raon-2013-0001

**Published:** 2013-02-01

**Authors:** Zhong Hua Luo, Alex Chung, Gibok Choi, Yih Huie Lin, Huajin Pang, Barry T. Uchida, Dusan Pavcnik, Miran Jeromel, Frederick S. Keller, Josef Rösch

**Affiliations:** 1 Dotter Interventional Institute, Oregon Health & Science University, Portland, USA; 2 Department of Radiology, Xijing Hospital, Fourth Military Medical University, Xi’an, People’s Republic of China; 3 Clinical Institut of Radiology, University Clinical Center, Ljubljana, Slovenia

**Keywords:** experimental radiology, radioopaque experimental clot, stroke, preclinical interventional radiology, animal model

## Abstract

**Background:**

Barium sulfate powder used for radiopacity of experimental blood clots (EBCs) for testing mechanical thrombectomy devices (MTD) has negative effects on EBCs mechanical properties. *In vitro* and *in vivo* exploration was performed to determine if the iodine based contrast medium will have less negative effects on the EBCs than barium.

**Materials and methods:**

Fresh blood from 2 swine was used to create fibrinogen enhanced and thrombin initiated EBC in tubes. Iodine radiopacity was achieved by mixing the blood with 65% Iohexol or by soaking the EBCs for 2 or 24 hours in Iohexol. The EBCs opacified with barium served as controls*. In vitro* study: The EBCs were subjected to four tests, manual elongation, catheter injection, radiopacity and contrast wash out tests. *In vivo* study: The common carotid arteries of 2 swine were embolized by either barium EBC or EBC soaked for 24 hours in Iohexol. The duration of radiopacity of the different EBCs was compared.

**Results:**

The EBCs opacified with Iohexol initially had higher radiopacity than the barium opacified EBCs. However, their opacity rapidly decreased with saline soaking and, particularly, after they were embolized in live animals. The mechanical properties of Iohexol opacified EBCs were inferior to barium opacified EBCs. The Iohexol mixed EBCs were less firm and elastic and half of them fragmented during catheter injection. The Iohexol soaked EBCs exhibited decreased tensile strength and elasticity compared to the barium EBCs.

**Conclusions:**

Compared to barium, iodine based contrast medium does not offer any advantage for opacifying EBCs.

## Introduction

Radiopacity of experimental blood clots (EBCs) is an important feature for evaluation of mechanical thrombectomy devices (MTDs) used for treatment of acute stroke. Radiopacity of EBCs allows fluoroscopic visualization during experimental embolization in an animal and direct observation of the MTD performance including fragmentation of the EBCs. Radiopacity of EBCs for the treatment of stroke with MTDs has been achieved by adding and carefully mixing barium sulfate powder with blood.^1^–^3^ Chueh *et al.*, however, showed that the presence of barium sulfate significantly reduces the EBCs elasticity.^2^ They also investigated using tantalum and tungsten silicic acid for EBC radiopacity, but found them unsuitable for testing MTDs.^2^ We explored the iodine based contrast agent, Iohexol, to opacity EBCs for both *in vitro* and *in vivo* studies to determine if it provides sufficient radiopacity and has less negative effects than barium sulfate on the mechanical properties of the EBCs.

## Materials and methods

The research was approved by the Institutional Care and Use Committee. Two swine weighing 45 and 42 kg were used as donors and blood was obtained from their femoral arteries. The animals underwent standard general anesthesia with intubation and artificial ventilation as described previously. 4,5 Hemostasis was achieved as described previously.^6^ After exposure of the right femoral artery, a 7 F sheath was introduced to obtain blood for EBC formation. The animals were then recovered and brought back the next day for embolization of the EBCs into their common carotid arteries. After embolization, x-ray of the EBCs and selective angiographic follow-up, the animals were euthanized. The procedures were done in an angiographic room equipped with GE-OEC 9800 cardiac mobile system with digital imaging (GE Medical System, OEC, Salt Lake City, UT).

### Clot formation

The model used for testing was a fibrinogen enhanced, thrombin initiated EBC that had been formed in a tube.^3^ Sixty five percent Iohexol (Omnipaque 300, GE Health Care Inc., Princeton, NJ) was used to opacify the EBC. Two techniques of Iohexol opacification were explored, mixing and soaking. With the *mixing technique*, 2 ml of Iohexol were added to 200 mg of bovine fibrinogen powder (Calbiochem, La Jolla, CA) and mixed well with 10 ml of blood in between two 12 ml syringes connected with a 3-way stopcock. Twenty five IU of bovine thrombin (Gen Trac, Inc., Middleton, WI) were then added to the mixture and carefully flushed about 10 times between the syringes. The final mixture was then injected into an 80 cm long polyvinyl chloride (PVC) tube with inner diameter of 4 mm. With the *soaking technique*, EBCs were created by mixing 200 mg of fibrinogen with 10 ml of blood and then with 25 IU of thrombin and injected into a PVC tube. EBCs initiated by 200 mg fibrinogen and 25 IU thrombin and made opaque by addition of 1 gr of barium sulfate powder (Spectrum Quality Products Inc., Gardena, CA) served as a control for comparison.^3^ The filled tubes were kept at room temperature for 1 hour. The EBCs produced with these techniques were then flushed into a basin filled with saline and cut into 2 cm long pieces. The EBCs formed by mixing technique (Mixs) and the control barium opaque EBCs (Bas) were immediately tested. The EBCs formed by soaking technique were first placed into a basin containing 65% Iohexol. These EBCs were tested after soaking in Iohexol for either 2 hours (2hSs) or 24 hours (24hSs). The EBCs soaked for 24 hours were kept soaking in a basin that was refrigerated at 4°C.

### *In vitro* study

*Clot testing.* The EBCs were subjected following four tests, manual elongation, catheter injection, opacity and contrast wash out tests. Twenty EBCs were tested in each group, ten from each animal.

*Manual elongation test.* The manual elongation test measuring the EBC tensile strength was performed in a room temperature saline solution.^3^ Both ends of the 2 cm long EBC were grasped with forceps and slowly stretched until the EBC fragmented. The length of stretching was measured with a ruler and recorded. A low tensile strength EBC usually fragmented well before its length was doubled. A firm EBC withstood stretching to almost double its original length.

*Catheter injection test.* The catheter injection test was done to evaluate the EBCs’ elasticity and to determine if the EBCs were able to withstand injection through a long, small diameter sheath.^3^ Tested EBCs were 4 mm in diameter. They were aspirated into a short plastic tube and injected with a saline filled 20 mL syringe through a 90 cm long 8F (2.2 mm inner lumen) Flexor-Shuttle Select introducer set (Cook Medical, Bloomington, IN) onto a smooth white surface. The size, shape and the degree of fragmentation of the EBC after injection were recorded by photography. The EBC’s length and diameter were measured before and after injection.

*Radiopacity evaluation.* For evaluation of radiopacity, ten pieces of 2 cm long EBCs were placed in a Petri dish. A small tube containing 6% barium suspension was added for reference. Digital images of EBCs before and after injection through the catheter were obtained on the cardiac mobile system with a standard brightness of 50 and contrast of 54. The relative mean gray values of EBCs in the digital images were then measured using the Image J software (NIH, Bethesda, MD). These values were converted into adjusted opacity percentages where 100% indicated the opacity of the reference barium tube.

*Radiopacity duration test – “a washout test”.* For evaluation of the radiopacity duration, the EBCs were immersed into room temperature saline. After soaking for 30 minutes, 60 minutes and 90 minutes their digital images were obtained to evaluate their loss of radiopacity. The manual elongation test was also performed on the washout EBCs.

### *In vivo* studies

Embolization of EBCs into the common carotid artery (CCA) was done to evaluate their mechanical properties, *i.e.* fragmentation and the degree and duration of their radiopacity. After EBC creation, a 90 cm long 8 F Flexor-Shuttle Select Introducer Set with inner lumen of 2.2 mm was introduced via the femoral artery and baseline arteriograms of both CCAs were obtained. In both animals, a 3 cm long piece of barium EBC was injected into the right CCA and a 3 cm long EBC formed with 24 hour soaking was injected into the left CCA. Radiographs of the neck and head were obtained immediately and 30, 60 and 90 minutes after injection. They were evaluated for homogenicity and radiopacity of the injected EBCs.

## Results

**Figure 1.**
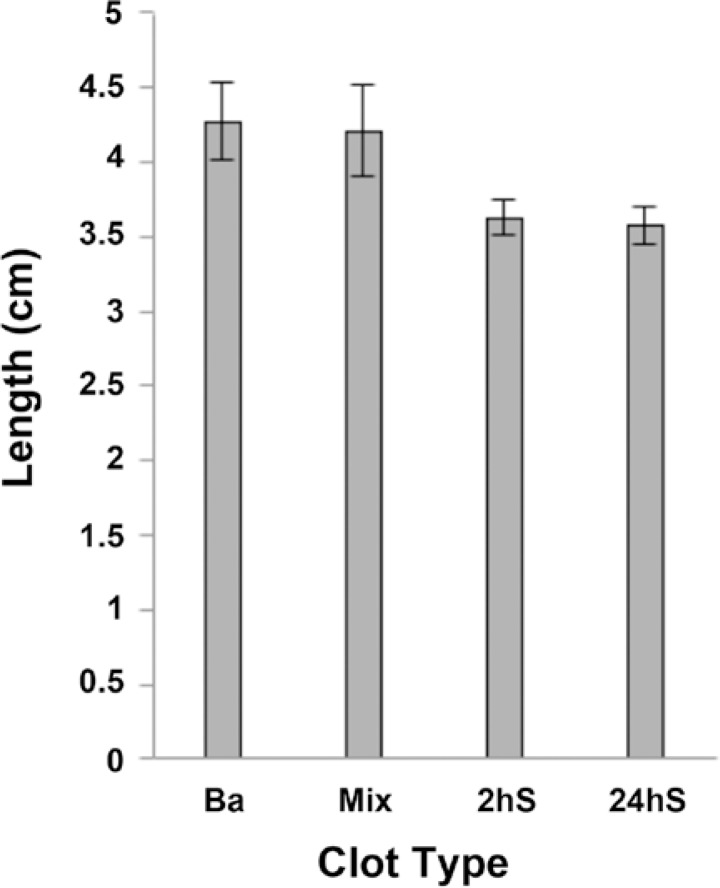
Results of manual elongation tests for four types of EBCs with starting length of 2 cm. The bar length indicates the average maximum elongation of 20 tested EBCs. Ba – control EBC with barium, Mix – EBC obtained by mixing with Iohexol, 2hS and 24hS – EBCs obtained by soaking in Iohexol for 2 hours and 24 hours respectively.

All EBCs formed well within 60 minutes and had dark red color. With the Bas, the color was slightly lighter, particularly after catheter injection. During the manual elongation test, the Mixs broke at a mean of 4.19 cm (SD± 0.25 cm) (Figure 1). Similarly, the Bas broke at median of 4.27 cm (SD± 0.22 cm). The 2hSs broke at a mean of 3.59 cm (SD± 0.13 cm) and 24hSs at a mean of 3.63 cm (SD± 0.16 cm). All soaked EBCs retained their tensile strength during 30, 60 and 90 minutes saline soaking and broke with a mean difference of less than 0.1 cm.

**Figure 2.**
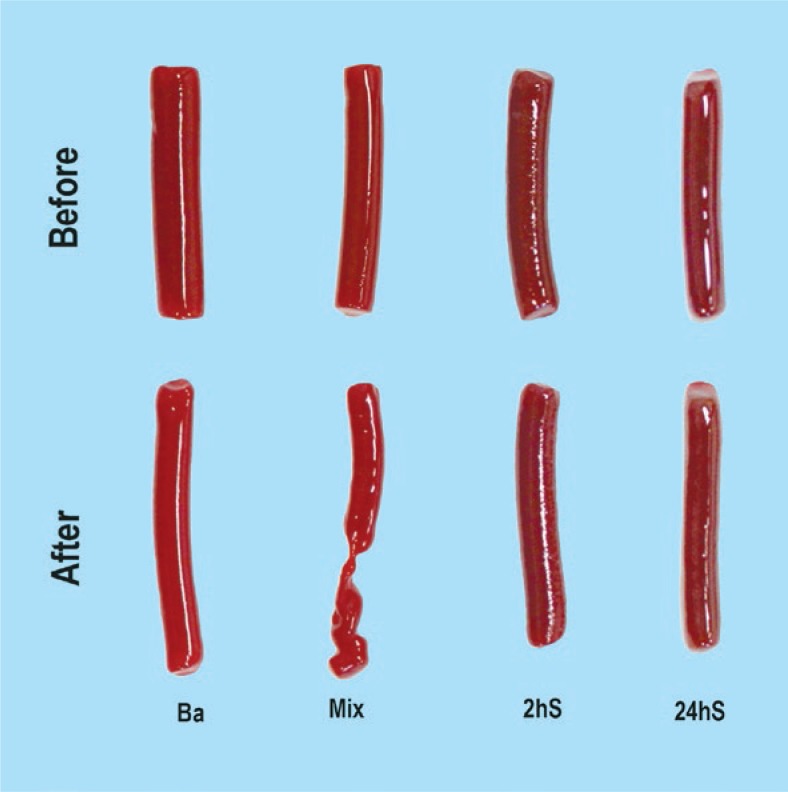
Examples of catheter injection tests of four types of EBCs. Ba – control EBCs with barium, Mix – EBCs obtained by mixing with Iohexol, 2hS and 24hS – EBCs obtained by soaking in Iohexol for 2 hours and 24 hours respectively.

With the catheter injection test, the Mixs exhibited deformity and fragmentation in 10 of 20 tested clots (Figure 2). This was similar for all three times of these EBCs soaked in saline. The unbroken Mixs became elongated by a mean of 0.2 cm (SD± 0.05 cm) and narrowed by a mean of 0.7 mm (SD ±0.04 mm) after injection. The Bas, the 2hSs and 24hSs did not exhibit any deformity or breakage after catheter injection (Figure 2). The elongation and narrowing for Bas was a mean of 0.53 cm (SD ± 0.21 cm) and a mean of 0.25 mm (SD ±0.12 mm), respectively. The elongation and narrowing of 2hSs was a mean of 0.12 cm (SD ± 0.12 cm) and a mean of 0.41 mm (SD ± 0.01 mm), respectively. The 24hSs elongated at a mean of 0.21 cm (SD ± 0.02 mm) and narrowed at a mean of 0.42 mm (SD ± 0.01 mm) after catheter injection (Figure 2).

**Figure 3.**
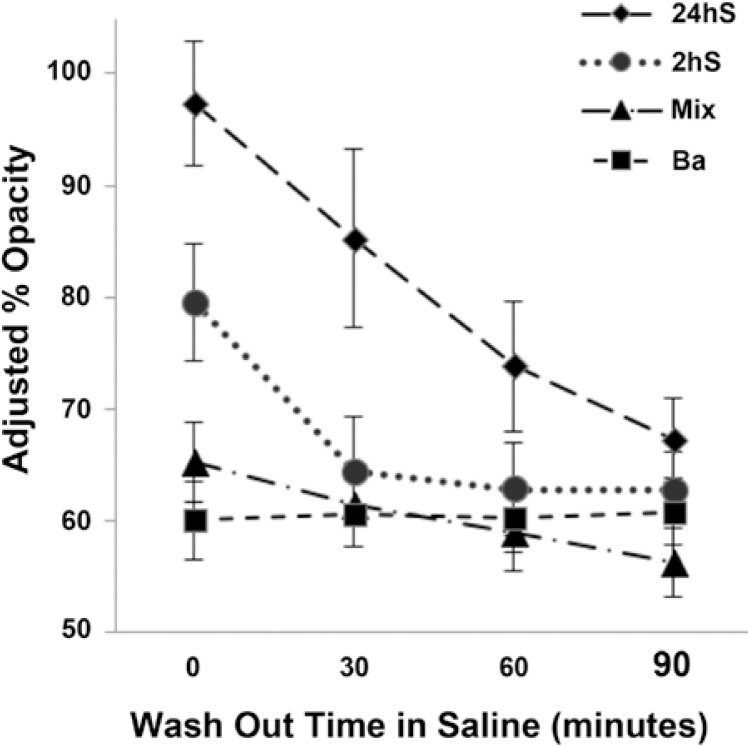
Results of radiopacity and its duration tests in four types of EBCs. Ba – control EBCs with barium, Mix – EBCs obtained by mixing with Iohexol, 2hS and 24hS. EBCs obtained by soaking in Iohexol for 2 hours and 24 hours respectively. The radiopacity of Iohexol EBCs is initially high, but rapidly decreases. The radiopacity of barium EBC is low, but stable.

Initial radiopacity of the Mixs was a mean of 64.2% (SD ±5.2%) and decreased after saline soaking gradually to a mean of 55% (SD ±4.3%) at 90 minutes (Figure 3). The Bas exhibited initial opacity of a mean of 58% (SD ± 4.2%) without changes with saline soaking. The 2hSs had initial opacity of a mean 79.5% (SD ± 5.2%) that gradually decreased to a mean of 62.6% (SD ± 3.3%) after 90 minutes of saline soaking. After catheter injection, they exhibited a decrease in opacity of a mean of 5.5% (SD ± 0.5%). The 24hSs had initial mean opacity of 97.3% (SD ± 5.6%) that decreased to a mean opacity of 67.1% (SD ± 3.4%) after 90 minutes of saline soaking. The opacity decrease after injection for 24hSs was a mean of 3.75% (SD ± 3.7%).

**Figure 4.**
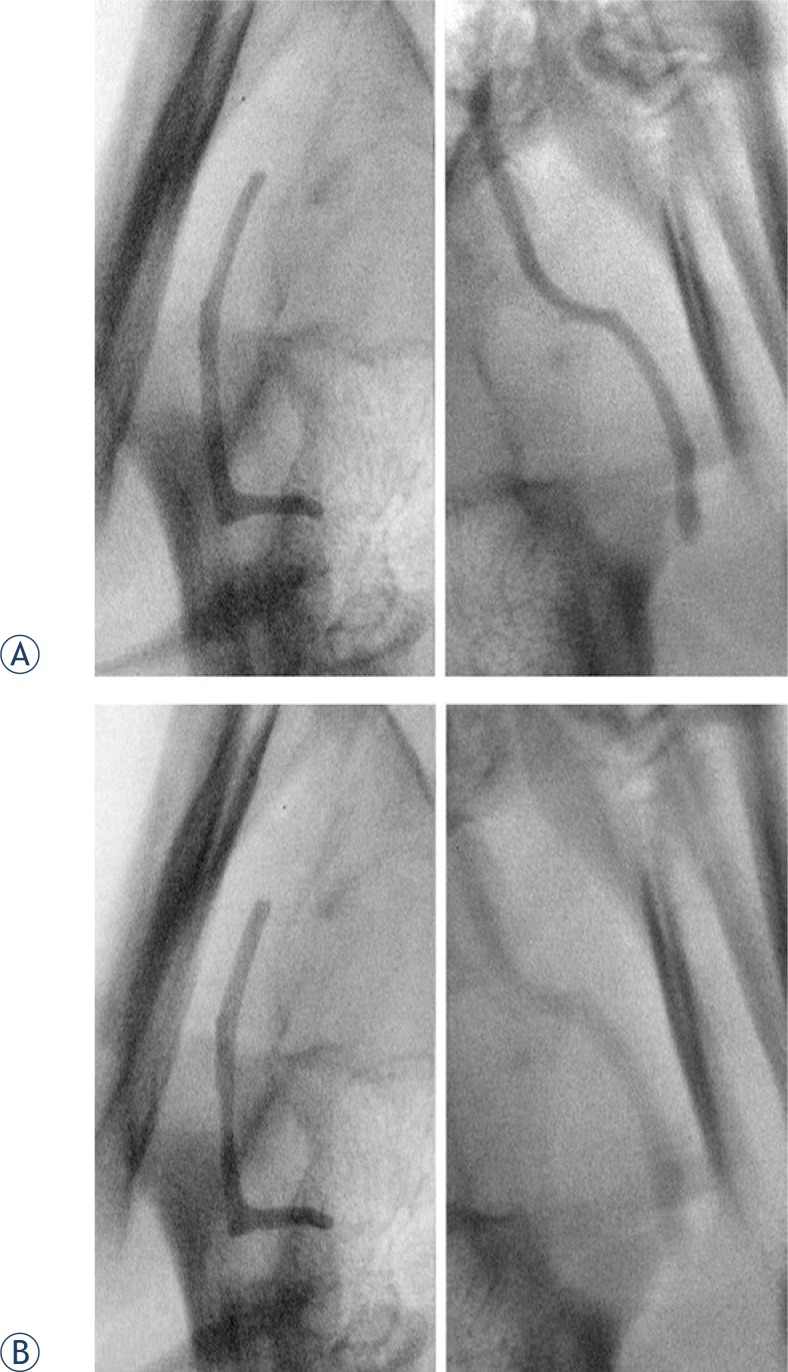
Close up radiographs of EBCs embolized into the common carotid arteries (CCA) of a live swine. Barium EBC is in the right CCA, 24 hour Iohexol soaked EBC is in the left CCA. (A) Initial radiographs show similar opacity of both EBCs. (B) Radiographs 60 minutes after embolization show no radiopacity change of the barium EBC and significant decrease of radiopacity of the Iohexol EBC.

With embolization of EBCs in the CCAs of live animals, the Mixs had moderate opacity. However, their opacity rapidly decreased after embolization and by 90 minutes was only minimal. The barium EBCs retained good opacity for 3 hours (Figure 4). Selective arteriographies of CCAs done at the end of studies revealed CCA occlusions.

## Discussion

We used the EBC model formed with 200 mg fibrinogen and 25 IU thrombin plus 1 g barium sulfate powder as the control because we found it sufficiently firm and elastic in our previous work.^3^ We are using it for testing new MTDs and for hands-on courses instructing physicians on MTDs use. Two types of EBC opacification (mixing and soaking) with the iodine based contrast material, Iohexol were explored. With the mixing technique, 2 ml of Iohexol was used because 1 ml did not give sufficient opacity. Both the Mixs clots and the Bas clots were available in 1 hour as acute EBCs. The 2hSs clots can also be used in the same session. These differ from the 24 hSs, made using the Ponomar et al technique that are less practical because they require two days to be available for testing.^7^

Results of this study did not show an advantage to opacifying EBCs by iodinated contrast material compared to barium. On the contrary, the mechanical properties and duration of radiopacity of Iohexol EBCs were inferior to barium opacified EBCs making them less suitable for MTD testing. The tensile strength of Iohexol mixed EBCs is similar to that of the barium EBCs. However, they are less firm and elastic and a high percentage fragments during catheter injection. The Iohexol soaked EBCs don’t break during catheter injection, but have lesser tensile strength and elasticity than the barium EBC model. The initial opacity of Iohexol EBCs is greater than the barium EBCs, particularly, those prepared with 24 hour soaking. However, this is only short lasting and rapidly decreases with saline soaking. The decrease in radiopacity of Iohexol EBCs in live animals is faster than during saline soaking, undoubtedly due to fast absorption of Iohexol. On the other hand, barium EBCs don’t have loss of opacity during saline soaking or as thromboemboli in living animals. Post mortem examination of these EBCs revealed the barium powder is well mixed with red cell clumps and fibrin.^3^

In conclusion, iodine based contrast medium has greater negative effects on the mechanical properties and radiopacity of EBCs than barium sulfate powder. Barium tube EBCs enhanced by fibrinogen and initiated by thrombin are preferable for MTD testing.
